# Study on the Tip Leakage Loss Mechanism of a Compressor Cascade Using the Enhanced Delay Detached Eddy Simulation Method

**DOI:** 10.3390/e26040295

**Published:** 2024-03-28

**Authors:** Shiyan Lin, Ruiyu Li, Limin Gao

**Affiliations:** 1 School of Power and Energy, Northwestern Polytechnical University, Xi’an 710072, China; lsy960313@mail.nwpu.edu.cn; 2 School of Aerospace Engineering, Xi’an Jiaotong University, Xi’an 710049, China

**Keywords:** compressor, tip leakage flow, enhanced delay detached eddy simulation, tip region loss, the entropy-generation rate

## Abstract

The leakage flow has a significant impact on the aerodynamic losses and efficiency of the compressor. This paper investigates the loss mechanism in the tip region based on a high-load cantilevered stator cascade. Firstly, a high-fidelity flow field structure was obtained based on the Enhanced Delay Detached Eddy Simulation (EDDES) method. Subsequently, the Liutex method was employed to study the vortex structures in the tip region. The results indicate the presence of a tip leakage vortex (TLV), passage vortex (PV), and induced vortex (IV) in the tip region. At i=4°,8°, the induced vortex interacts with the PV and low-energy fluid, forming a “three-shape” mixed vortex. Finally, a qualitative and quantitative analysis of the loss sources in the tip flow field was conducted based on the entropy generation rate, and the impact of the incidence on the losses was explored. The loss sources in the tip flow field included endwall loss, blade profile loss, wake loss, and secondary flow loss. At i=0°, the loss primarily originated from the endwall and blade profile, accounting for 40% and 39%, respectively. As the incidence increased, the absolute value of losses increased, and the proportion of loss caused by secondary flow significantly increased. At i=8°, the proportion of secondary flow loss reached 47%, indicating the most significant impact.

## 1. Introduction

The tip leakage flow, as one of the most important secondary flows in the compressor, has a significant impact on the efficiency [1], and it accounts for approximately 20∼30% of the total compressor loss [2]. Due to the pressure gradient between the pressure and suction surface, the leakage flow ejects from the tip clearance into the adjacent passage, shears, and mixes with the mainstream, forming the tip leakage vortex (TLV). Under the coupling of multiple boundary layer interference on surfaces such as the endwall and blade [3], strong shear between the mainstream and leakage flows [4], and the interaction of various vortex structures [5], the compressor tip region exhibits strong three-dimensional, nonlinear, and multi-modal complex flow characteristics [6,7]. Hence, thoroughly exploring the correlation mechanism between losses and complex flow structures in tip region is a prerequisite for the fine design and effective flow control of the modern compressor, holding significant engineering significance.

With the advancement of computers and numerical simulation methods, research on tip loss has transitioned from early model-based assumptions [8,9], regular exploration of tip geometry and aerodynamic parameter variations [10,11,12] into the correlation mechanisms between the detailed flow structures and loss. In the study of a low-speed compressor cascade, Han [13] indicated that the interaction between the TLV and the Passage Vortex (PV) is the main source of loss in the tip region of the cascade. Yu [14,15] and Huang [16], in separate studies on a large low-speed axial compressor and a compressor cascade with upper wall movement, respectively, both found that in the near-stall condition, there is a corner vortex (CV) in the tip region. The interaction between CV and the TLV is identified as a significant factor leading to increased losses. Moghadam [17] suggested that the induced vortex (IV) generated inside the passage accelerates the breakdown of the TLV under off-design conditions, resulting in the decreasing efficiency. It can be seen that there is still no consensus on the sources of loss from the perspective of detailed structure in the tip region, making it an open issue worthy of further investigation.

One of the reasons leading to the controversy in the sources of tip loss is the insufficient fidelity of the numerical methods employed. The Reynolds-Averaged Navier–Stokes (RANS) method and Unsteady Reynolds-Averaged Navier–Stokes (URANS) method lack accuracy in predicting flow phenomena characterized by strong shear, such as tip leakage, especially in extreme conditions [18]. In contrast, although the large eddy simulation (LES) has been proven to have high accuracy in tip flow fields, the requirements of the computational cost currently prevent its application in the industrial domain [19]. The DES-series method [20,21,22], as a type of RANS/LES coupling method, has been widely applied for predicting the flow in the large separation flow in the compressors [23], especially in the hub corner [24,25,26,27] and the tip region [6,18,28]. It combines the advantages of RANS, which requires fewer grids near the wall, and LES, which accurately predicts flow in large separated regions. As a result, it achieves a balance between computational cost and accuracy, offering the possibility of high-fidelity numerical simulations for industrial applications. However, DES-series methods lack a mechanism for modeling the conversion of the modeled turbulent kinetic energy (TKE) to resolved TKE near the interface between the RANS and LES domain, resulting in an inevitable gray area [20,21,22]. The gray area exists in the form of a slow LES development (SLD) problem when encountering the “multiple boundary layer attachments + shear effect” typical in the case of tip leakage flow. In this situation, fluctuation cannot be resolved at the initial shear region, thereby affecting the accuracy of downstream predictions [29]. Research indicates that DES97, DDES, and IDDES methods all suffer from inaccuracies in predicting aerodynamic parameters [28], detailed structures [6], unsteady fluctuations [30], and TKE [18] in the prediction of compressor tip flow.

In response to this issue, Deck S. [31] and Shur [32] proposed the Zonal Detached Eddy Simulation (ZDES) method and the Enhanced Delay Detached Eddy Simulation (EDDES) method, respectively. They considered the local vortex characteristics and modified the characteristic scale of the dissipation term in the original DDES method, reducing the eddy viscosity and enhancing the resolution capability of the initial shear layer. Riera [33] employed the ZDES and URANS methods for numerical predictions of the first-stage transonic rotor in CREATE. The results indicated that although ZDES demonstrated good agreement with experiments in terms of predicting time-averaged parameters, its ability to resolve fluctuations near the initial shear layer was comparable to URANS. He [6] compared the application of the original DDES and EDDES methods on a low-speed, large-scale compressor rotor. The results showed that the EDDES method provided more accurate capture of the TKE and turbulence anisotropy.

Based on the EDDES method, high-fidelity simulations of unsteady tip clearance flow could be obtained. Therefore, it is essential to utilize methods capable of describing the contributions of unsteady features to analyze tip losses accurately. However, traditional analyses of losses in turbomachinery often rely on parameters such as entropy or total-pressure-loss coefficients calculated based on the averages of parameters like the total temperature and total pressure. It is difficult to separately distinguish the losses caused by unsteady fluctuations from the overall losses through these parameters, making it challenging to precisely quantify the contribution of mixing and interference between different structures. Furthermore, these parameters can only be used to indicate the energy reduction of the fluid from the inlet to the computational location, representing cumulative loss effects. They are unable to establish a correlation between local flow structures and the ability to generate losses [34]. Li [34], Yamada [35], Deveaux [36] quantified the losses in the corner and tip region of the compressor using the entropy generation rate, determining the loss generation capabilities of the different flow structures.

In summary, obtaining a high-fidelity flow field with unsteady characteristics is a primary requirement. Based on this, the use of entropy-generation-rate analysis, taking into account local unsteady loss, is more conducive for studying the loss mechanisms in the tip region. Therefore, in this study, the EDDES method has been conducted for a high-load cantilevered stator cascade of different incidences. Based on high-fidelity numerical results, the Liutex method was used to study the vortex structures in the tip region. Subsequently, the entropy generation rate was used to quantify the tip flow loss in the unsteady flow field. And the associated mechanisms between high-loss sources and detailed structures of different incidences were established.

## 2. Model and Method

### 2.1. Compressor Cascade NPU01

**Figure 1:**
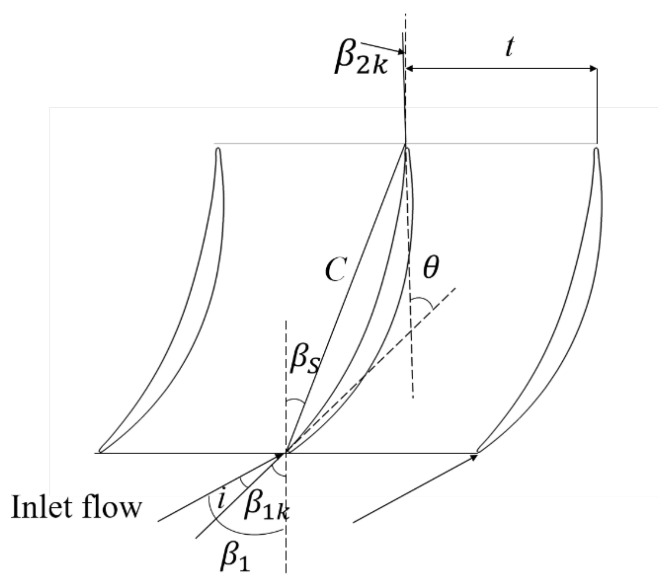
Two-dimensional cascade configuration of NPU01.

A high-loading compressor cantilevered stator cascade, NPU01, was selected to study the loss caused by the tip leakage flow. The main geometric parameters of the cascade are shown in Table 1, and the schematic diagram is illustrated in Figure 1. The blade height is 99 mm while the tip clearance is 1 mm (1.53%C). The cascade stagger angle χ is 21.27°, while the blade inlet angle $\beta_{1k}$ and blade outlet angle $\beta_{2k}$ are 47.08° and −1.98°, respectively, causing the camber angle of the blade to be 49.16°. In both experimental and numerical instances, the incident flow is adjusted by varying the inlet flow angle. The design inlet Mach number is 0.5 with an inlet chord-based Reynolds number of approximately $7.7\times 10^{5}$, while the design incidence is 0°. In this paper, the incidences are 0°, 4° and 8° and the investigation explores the influence of the incoming incidence on the tip flow field structure and loss mechanism. The diffusion factors *D* of 0°, 4°, and 8° are 0.442, 0.518 and 0.546, respectively. The diffusion factors are obtained from Equation (1), where $w_{1},w_{2},\beta_{1},\beta_{2},\Delta w_{u},\tau$ represent the inlet velocity, outlet velocity, inlet flow angle, outlet flow angle, twist velocity, and solidity, respectively.

(1) $$D=1-\frac{w_{2}}{w_{1}}+\frac{\Delta w_{u}}{2w_{1}\tau }$$

To validate the simulation, the experiment was performed on a continuous high-speed subsonic wind tunnel in the National Key Laboratory of Aerodynamic Design and Research of the Northwestern Polytechnical University [37], which houses a linear compressor cascade with 8 blades. The flow from the contraction entered the upstream part of the test section, a rectangular duct with a cross-section 100 mm high by 300 mm wide. No specific control measures were implemented on the endwall boundary layer, and according to the Ref. [38], the endwall boundary layer was approximately 12 mm thick at the leading edge of the blade. No boundary layer trip was applied to the blade’s surface. The oil flow experiments [5] and the outlet Mach number distribution were adopted in order to validate the simulation accuracy [39,40]. The inlet and outlet boundary conditions are summarized in Table 2.

### 2.2. Enhanced Delay Detached Eddy Simulation (EDDES) Method

Compared to other RANS/LES coupling methods, DES-series methods have simple structures that are easy to implement. For example, the SA model [20] (see Equation (2)) achieves the transition from RANS to LES regions by modifying the original distance $d_{w}$ in the dissipation term, which is the distance between the grid center and the wall, to a characteristic length, denoted as $\tilde{d}$.

(2) $$\frac{D\tilde{\nu }}{Dt}=\overset{\mathrm{production}}{\overset{︷}{c_{b1}\tilde{S}\tilde{\nu }}}+\overset{\mathrm{diffusion}}{\overset{︷}{\frac{1}{\sigma }\left[\nabla \cdot \left(\left(\nu +\tilde{\nu }\right)\nabla \tilde{\nu }\right)+c_{b2}{\left(\nabla \tilde{\nu }\right)}^{2}\right]}}-\overset{\mathrm{dissipation}}{\overset{︷}{c_{w1}f_{w}{\left[\frac{\tilde{\nu }}{\tilde{d}}\right]}^{2}}}$$

(3) $$\tilde{d}=\min\left(d_{w},C_{des}\max\left(\Delta x,\Delta y,\Delta z\right)\right)$$

**Figure 2:**
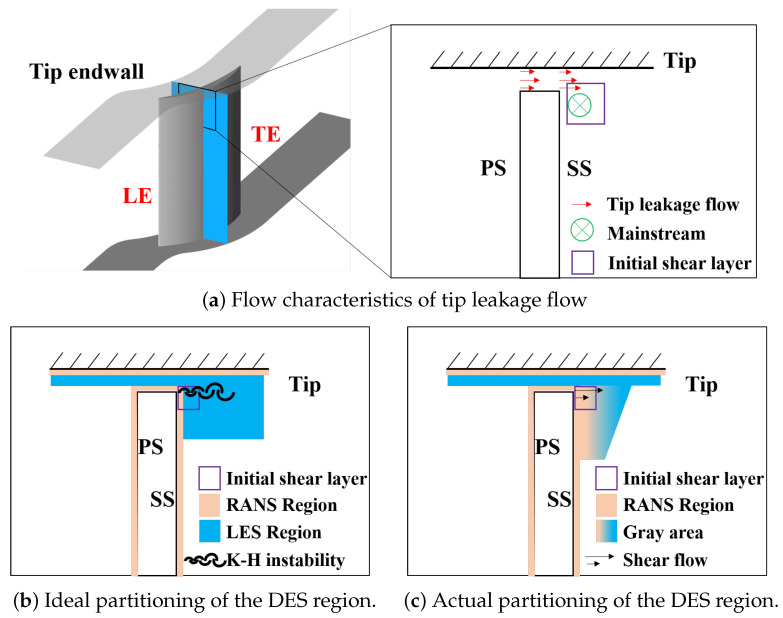
The diagram of the impact of the gray area on tip leakage flow.

However, DES-series methods lack a mechanism for modeling the conversion of modeled TKE to resolved TKE near the interface between RANS and LES, resulting in an inevitable gray area. Even though Spalart [22] proposed an improved version, DDES, in 2006 to address the issues caused by Modeled Stress Depletion (MSD) by delaying the development of LES, this version is only applicable to corner separation rather than tip leakage in compressor applications. This is because the unique flow structure of tip leakage flow presents new challenges for the DDES method [29]. As shown in Figure 2a, there is a flow along the tangential direction from the tip clearance in the compressor, forming a confined jet that shears with the mainstream. The mechanism of the gray area’s impact in such special flows is illustrated in Figure 2c. In the region where the mainstream initially shears with the leakage flow and should promptly transition to LES (as shown in Figure 2b), the transition is delayed due to the influence of the SLD problem caused by the gray area. This results in an excessively strong modeled eddy viscosity in the initially sheared region, suppressing the generation of Kelvin–Helmholtz (K-H) instability, delaying the instability of the shear layer, impeding the generation, development, and breakdown of vortices. Consequently, this underestimates the separation of the flow.

In response to this issue, Shur [32] proposed an EDDES method based on a Vortex Tilting Measure (VTM). This method could reduce the eddy viscosity at the separation initiation shear location, promoting the instability and development of the shear layer.

Initially, it modified the sub-grid scale in the original DDES method by:

(4) $${\tilde{\Delta }}_{\omega }=\frac{1}{\sqrt{3}}\max_{n,m=1,8}\left|{\vec{n}}_{\omega }\times \left({\vec{r}}_{n}-{\vec{r}}_{m}\right)\right|$$

where ${\vec{n}}_{\omega }$ represents the unit vector along the local vorticity axis, and ${\vec{r}}_{n}$ represents the displacement vector between any two grid vertices. This can reduce the characteristic scale of the corresponding grid at the quasi-two-dimensional shear location during the initial separation. As a result, it reduces eddy viscosity, promoting the instability of the shear layer. Furthermore, Shur multiplied the new sub-grid scale by a flow-field detection function capable of distinguishing between quasi-two-dimensional shear and fully developed three-dimensional turbulence. The detection function is dependent on the VTM sensor. Its expression is as follows:

(5) $$F_{KH}\left(VTM\right)=\max\left\{F_{KH}^{\min},\min\left[F_{KH}^{\max},F_{KH}^{\min}+\frac{F_{KH}^{\max}-F_{KH}^{\min}}{a_{2}-a_{1}}\left(VTM-a_{1}\right)\right]\right\}$$

(6) $$VTM=\frac{\sqrt{6}\left|\left(\hat{S}\cdot \vec{\omega }\right)\times \vec{\omega }\right|}{\omega^{2}\sqrt{3\mathrm{tr}\left({\hat{S}}^{2}\right)-{\left[\mathrm{tr}\left(\hat{S}\right)\right]}^{2}}}$$

where the coefficients $F_{KH}^{\max}$, $F_{KH}^{\min}$, $a_{2}$ and $a_{1}$ are equal to 1, 0.1, 0.3, and 0.1, respectively. When the eigenvalue tensor of the shear strain rate tensor $\hat{S}$ is correlated with the local vorticity vector, i.e., $\vec{\omega }$, fluid deformation occurs in a plane perpendicular to the local vorticity vector, the $|\left(\hat{S}\cdot \vec{\omega }\right)\times \vec{\omega }|$ approaches 0 while the VTM value approaches 0. This indicates that the fluid deformation at this position is quasi-two-dimensional, implying it takes place at the initial shear location. On the contrary, when the fluid exhibits fully developed three-dimensional turbulence, $\hat{S}$ is uncorrelated with the local vorticity vector $\vec{\omega }$, and the value of the VTM approaches 1.

Finally, by using a piece-wise function, $F_{KH}\left(VTM\right)$, this method achieves a sub-grid scale close to $0.1{\tilde{\Delta }}_{\omega }$ in the initial shear region and the original sub-grid scale in the region of fully developed turbulence. This achieves the purpose of reducing the eddy viscosity in the initial shear region while ensuring that the region of fully developed turbulence remains at a normal level, improving the accuracy the simulated flow in the compressor tip region.

### 2.3. Computational Settings and Grid

In this study, the open-source Stanford University Unstructured (SU2) code [41], which is particularly well suited for aerodynamic problems [42] is employed to solve the Navier–Stokes (NS) equations. The upwind Roe scheme with MUSCL reconstruction is utilized to calculate the convective fluxes for the NS equations to achieve the second order in space. To regulate dissipation within the LES region of the flow, an adapted variant of the Roe scheme is incorporated into SU2 [43]. Beside, an upwind scheme is applied to discretize the convective fluxes in the SA equation. Regarding the time advancement, a dual-time stepping strategy with second-order implicit time integration is employed, and the physical time is set to $1\times 10^{-6}$ s, which is based on the suggestions in Ref. [44].

Regarding the boundary conditions, the inlet is specified with a uniform total temperature, and the total pressure is given with a radial distribution to achieve a boundary layer thickness of 10 mm in the incoming flow, consistent with the experiment. The inlet turbulence intensity is set as 1.5%, which is consistent with the experiment, while the inlet turbulent to laminar viscosity ratio is 10%. The outlet is set with an average static pressure. The incoming Mach numbers are $Ma_{1}=0.5$, leading the Reynolds number by $7.7\times 10^{5}$ based on the chord length.

**Figure 3:**
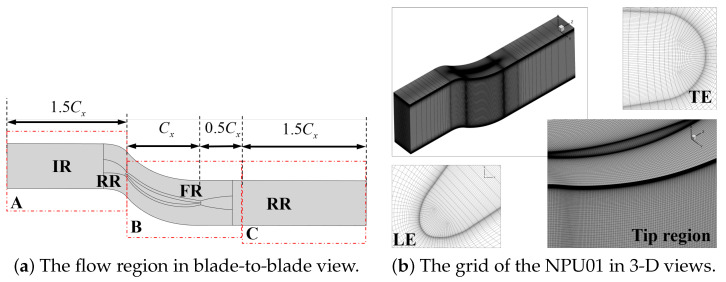
The diagram of the flow region and grid.

Due to the pronounced influence of the root corner separation at high incidence, this study conducts numerical simulations using a single passage with full blade height. Additionally, periodic boundary conditions are applied to both sides of the flow passage and the computational domain is illustrated in Figure 3. Structured O4H topology is employed for the main blade passage, and butterfly mesh topology is applied to model the blade tip gap. In the gap, there are 49, 25, and 353 layers in the spanwise, tangential, and streamwise directions, respectively. The wall distance of the first grid layer is $2\times 10^{-6}$ m, ensuring that $y^{+}<1$. According to the grid partition strategy, $x^{+}$ is less than 110 and $z^{+}$ is less than 100 in the focus region (FR region), i.e., the tip leakage and the corner separation area, which meets the requirements of the LES. The grid number is decreased to reduce the computation in the RR region, as shown in Figure 3a. In regions A and C, fewer grids are set, with particular emphasis on region B of the blade passage and extending the axial chord length (*C*) by 0.5 times from the outlet, resulting in a total grid number of 8.81 million. The grid distribution in different regions is shown in Table 3.

### 2.4. Local Entropy Generation Rate

This study, based on the entropy generation theory from the second law of thermodynamics, analyzes and studies the losses caused by various irreversible factors in the compressor blade tip region. Bejan [45] indicates that entropy generated in the flow due to two irreversible sources, viscosity and heat transfer, is the cause of flow field losses, as shown in Equation (7).

(7) $${\dot{S}}_{gen}={\dot{S}}_{visc}+{\dot{S}}_{therm}$$

As the temperature variation within the compressor is minimal, the entropy generation associated with the heat transfer could be neglected. The entropy generation from viscosity includes the time-averaged motion and the turbulence fluctuations, as shown in Equation (8). These two components represent the entropy increases caused by direct dissipation and turbulence dissipation, respectively.

(8) $${\dot{s}}_{gen}=\frac{1}{T}\left(2\nu S_{ij}S_{ij}+\tau_{ij}\frac{\partial u_{i}}{\partial x_{j}}\right)$$

where ν represents dynamic viscosity, while $S_{ij}$ is the strain-rate tensor of the mean velocity, indicating the direct entropy production rate based on the mean velocity. $\tau_{ij}=-\langle u_{i}^{\prime }u_{j}^{\prime }\rangle$ is the Reynolds stress tensor, while $\frac{\partial u_{i}}{\partial x_{j}}$ is the transient velocity gradient, representing the entropy increase due to turbulence fluctuations. The time averaging is performed on the results, while the window function is set as a Hann square. The duration of time averaging in this study is $1\times 10^{-2}$, comprising ten thousand physical time steps. The chosen duration for time averaging in this paper covers nearly twice the total fluctuation period of the entire flow field and 30 times the fluctuation period at the tip region.

**Figure 4:**
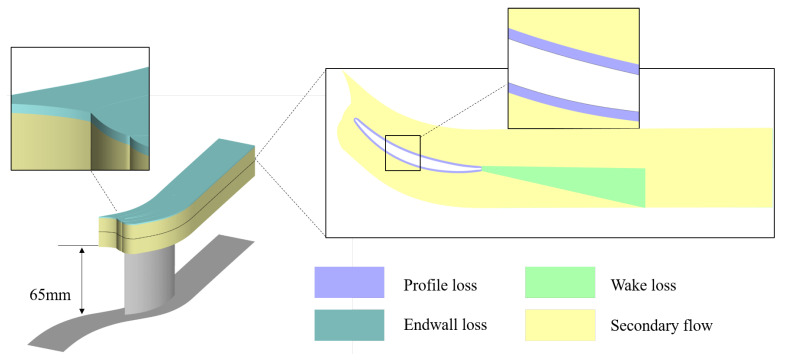
The schematic diagram of the integral regions for tip loss.

According to the second law of thermodynamics, the irreversible losses within a specified region per unit time are the product of the total entropy generation within the region and the ambient temperature. The integral formula is as follows:

(9) $$Loss=T_{0}\int_{V_{i}}{\dot{S}}_{gen}dV_{i}=T_{0}{\dot{S}}_{1}$$

where $V_{i}$ represents loss regions, and by integrating Equation (9) within each loss region, quantitative results for various types of losses can be obtained. Based on the overall flow characteristics of the flow field, the tip region, mid-span, and corner separation region are distinguished. Subsequently, according to the different flow phenomena in the tip flow field, as described in the literature [34,35], the loss regions in the blade tip region are divided into four parts, namely endwall loss, blade profile loss, wake loss, and secondary flow loss in the passage, as shown in Figure 4. In particular, regarding the losses caused by the endwall and blade profile boundary layers, since the entropy generation rate inside the boundary layer is much larger than outside (by more than two orders of magnitude), the division is based on the spatial distribution of the entropy-generation rate and the gradient of the standard entropy-generation rate of the solid surface. The thickness of the boundary needs to ensure complete coverage of the regions with significantly higher entropy generation rates near the blade surface (slight deviation outward of the integration region has little effect on the integration value of endwall and blade profile losses) [34]. The specific boundary layer thickness needs to be adjusted based on the contour of near-wall entropy generation rates for each operating condition. In this paper, a special definition is provided for the wake loss region, which differs from the approach described in Ref. [34] where the entire domain behind the trailing edge is categorized as the wake loss region. Only the vortex region formed by the boundary layers on the suction and pressure surfaces at the trailing edge is classified separately as a wake loss region, while the high entropy generation-rate region resulting from the downstream development of vortex structures inside the passage is attributed to secondary flow losses. As a result, the portion excluding endwall losses, blade profile losses, and wake losses is classified as a secondary flow loss region.

## 3. Results

### 3.1. The Accuracy of the EDDES Method in Tip Leakage Flow

**Figure 5:**
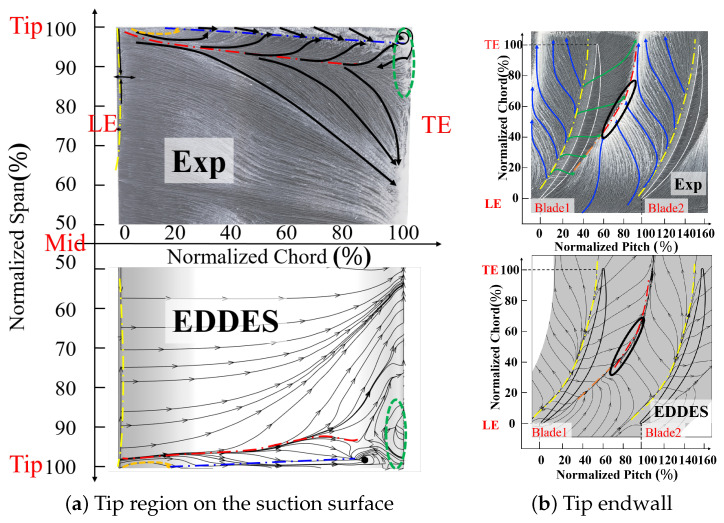
The diagram of the comparison between averaged streamline of the EDDES with the experimental oil flow pattern.

To validate the accuracy of the EDDES method, Figure 5 provides a comparison between the averaged streamline of the EDDES with the experimental oil flow pattern at $Ma_{1}=0.5,i=0{}^{\circ}$. Figure 5a shows the comparison of the suction surface at the blade tip region. The EDDES and experiments results exhibit good agreement at the LE separation and tip region of the suction surface. At the LE region, the yellow dashed line represents the LE separation line and it can be seen that the length of the leading-edge separation bubble is quite similar. At the tip region, the orange, red, and black dashed lines represent the position of the IV generation, the convergence line, and the separation line caused by the scraping of the TLV and the suction surface, respectively. At the TE region, the green dashed circle represents the recirculation region. It can be seen that they all correspond well in both spanwise and streamwise ranges, indicating that the EDDES method provides accurate predictions for the span range of the tip leakage vortex.

Figure 5b shows the comparison of the tip endwall. The separation lines on the pressure side of both align well, while on the suction side, the convergence lines in EDDES slightly deviate toward the passage. However, the overall trend is consistent, indicating that the EDDES method slightly overpredicts the tangential extent of the tip leakage vortex. Furthermore, the positions where the leakage vortex, passage vortex, and induced vortex interact with each other (indicated by the black circles in the Figure 5b, manifested as deflections in the streamlines and accumulation in the oil flow coatings), also correspond well.

Overall, based on the comprehensive comparison above, the EDDES method demonstrates good accuracy in predicting the tip leakage flow.

**Figure 6:**
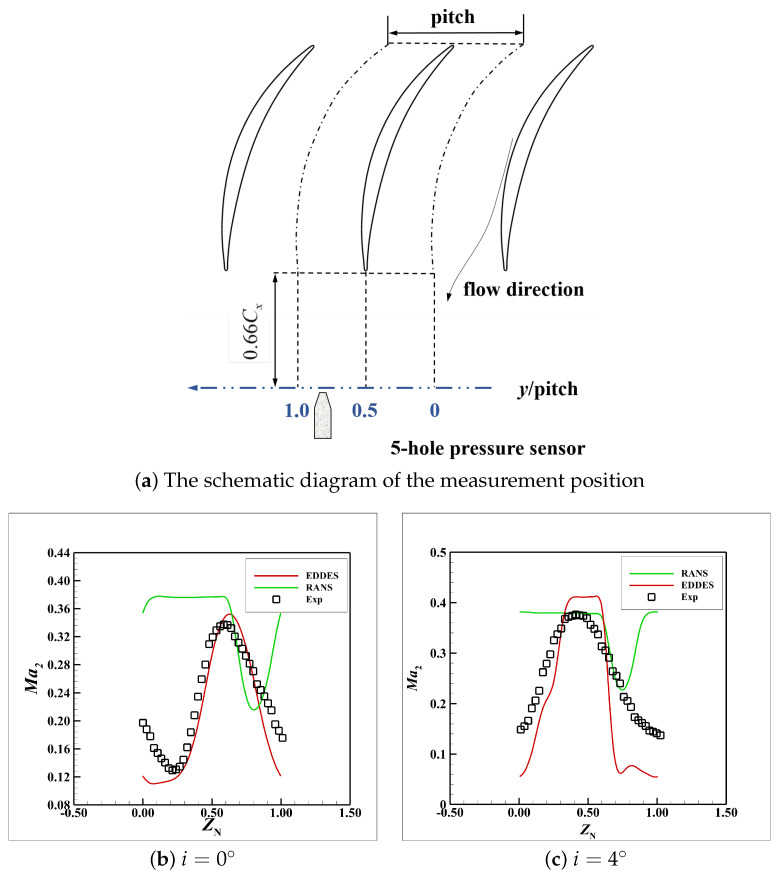
Comparison of the $Ma_{2}$ distributions between the experiment, RANS and EDDES results.

Figure 6b,c present the comparison of the distributions of the outlet Mach number, i.e., $Ma_{2}$ at i=0° and 4°, respectively. $X_{N}$ and $Z_{N}$ represent the normalized streamwise, and spanwise coordinates, respectively; see Equations (10) and (11).

(10) $$X_{N}=\frac{X}{C}$$

(11) $$Z_{N}=\frac{Z\tau }{C}$$

As shown in Figure 6a, the five-hole probe is located at the 166%*C* plane in the streamwise direction and at 85% span of the blade in the spanwise direction. It could be seen that the predictive accuracy of EDDES is higher than the RANS results in the tip region.

### 3.2. Flow Field Structure and Sources of Losses in Tip Region

#### 3.2.1. The Vortex Structure of the Tip Region

**Figure 7:**
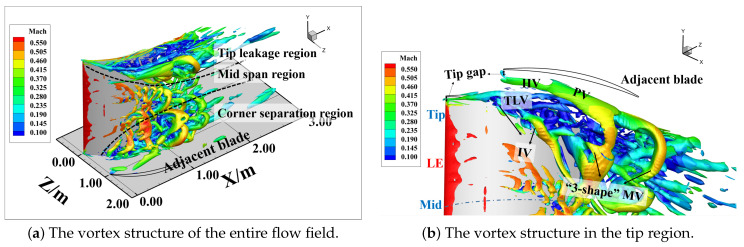
The transient vortex structure in the flow field(iso-surface $Liutex_{mag}=8000$ contoured by Mach number).

Figure 7 depicts the transient vortex structure in the flow field (iso-surface $Liutex_{mag}=8000$ contoured by Mach number). The Liutex method [46,47], as a third-generation vortex identification and visualization approach, possesses two distinct characteristics compared to second-generation criteria like *Q* criterion and $\lambda_{2}$ criterion: (I) It can effectively avoid the misidentification caused by wall shear contamination. (II) It is insensitive to threshold selection, ensuring the simultaneous display of strong and weak vortices in the flow field. In the internal flow field, such as the compressors, especially in the tip leakage flow characterized by multiple wall-affected boundary layers and strong shear effects, the Liutex method demonstrates significant advantages. It can be observed in Figure 7a that the entire flow field along the span exhibits three relatively independent vortex systems: the tip vortex system located above approximately 65% of the span, the separation in the mid-span, and the blade root corner separation below 40% range of the span.

For a detailed examination, Figure 7b provides a zoomed-in view of the blade tip area. It can be seen that there are four distinct vortex structures:

(1) Tip leakage vortex (TLV): Originating at the 3%C, it is initially located close to the suction surface before approximately 25%C. Subsequently, the trajectory of the tip leakage vortex turns into a passage, causing a reduction in fluid velocity around the vortex core. Low-energy fluid begins to appear on the inner side of the TLV near the suction surface tip. As the flow develops downstream, the low-energy fluid extends along both the spanwise and tangential ranges.
(2) Induced Vortex (IV): At the 25%C axial chord length, when the vortex core of the TLV begins to detach from the suction surface and deflect into the passage, the airflow on the inner side near the suction surface induces shear on the low-energy fluid near the wall. This induces the generation of the IV. The IV develops along the suction surface, and as the previous branch of IV is about to detach from the suction surface, a new branch begins to generate.
(3) The pressure surface branch of the horseshoe vortex (HV) near the leading edge of adjacent blades gradually merges with the passage vortex (PV) as it develops downstream.
(4) “3-shape” mixed vortex (“3-shape” MV): The IV, as it develops downstream, gets stretched and extends into the space. One leg of the IV, directed upwards, interacts with the low-energy fluid near the blade surface, while the other leg, pointing backward, merges with the PV. This interaction results in the generation of the “3-shape” mixed vortex (“3-shape” MV), so named due to its shape resembling the Arabic numeral 3, as depicted in the figure, covering the spanwise range from 65% to 100%. Eventually, it completely detaches from the blade suction surface at the trailing edge and mixes with the wake.

#### 3.2.2. Losses in Tip Region

**Figure 8:**
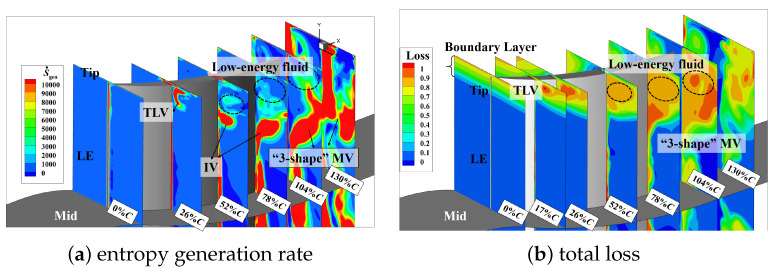
Entropy generation rate distribution and total pressure loss contour in the tip region at $Ma_{1}=0.5,i=4{}^{\circ}$.

Figure 8 shows the distribution of the entropy generation rate and total pressure loss in the tip region along the flow direction. Seven quasi-S3 sections are extracted from the leading edge to the trailing edge to analyze the distribution of high-loss structures (areas with a high entropy generation rate) and cumulative losses (areas with a large total pressure loss) at different streamwise positions. Through Figure 8a, it can be observed that before 20%*C*, the predominant contributor to losses in the tip region arises from the presence of the TLV. After that, the TLV deflects into the passage, forming IV and low-energy fluid near the suction surface. In the range from 20%*C* to 78%*C*, the IV gradually separates from the suction surface and subsequently converges with the low-energy fluid (indicated by the black circular dashed line in the Figure 8) and the PV, corresponding to regions of high loss in the flow field. Subsequently, it forms the “3-shape” MV and develops downstream. The high-loss region extends across the entire passage, reaching into the mid-blade region up to 65% of the blade span, eventually detaching from the trailing edge and blending with the wake.

The total pressure loss shown in Figure 8b represents the energy loss of the local position relative to the incoming airflow. Within the tip endwall boundary layer, the airflow experiences a reduction in velocity and a consequent decrease in energy due to viscous effects. The velocity of the fluid around the core of the TLV remains relatively low. Consequently, over the entire downstream range, the total pressure loss within the region covered by the TLV and its inner low-energy fluid is consistently high. Although the low-energy fluid has low velocity and insufficient energy, the level of momentum mixing induced by the fluctuations is limited, resulting in a relatively low loss-producing capability. Similarly, in the region where the “3-shape” MV begins to generate, the total pressure loss is not high. This is because the fluid velocity in this region is relatively high, and thus, there is no excessive loss of total pressure. In fact, the intense mixing between different vortex structures is the reason for the high-loss sources.

Therefore, it can be seen that, compared to total pressure loss, the entropy generation rate has more advantages when it comes to analyzing the source of the high-loss structures.

### 3.3. The Influence of the Incidences on Tip Leakage Flow

**Figure 9:**
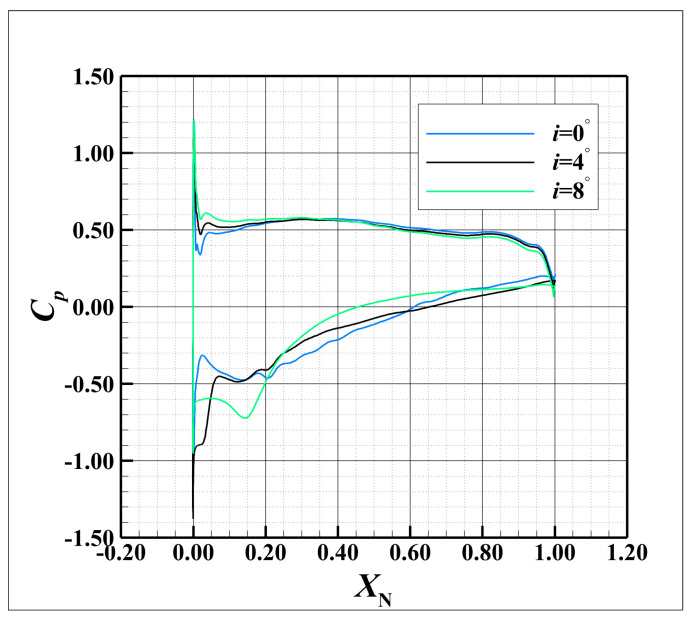
The static pressure coefficient distribution of the mid-span at i=0°,4°,8°.

The variation in the incoming incidence affects the initiation position of the compressor tip leakage, the trajectory of the tip leakage vortex, and the load distribution. Figure 9 shows the static pressure coefficient distribution at mid-span for EDDES-averaged flow fields at i=0°,4°,8°. It can be observed that with the increase in the incident angle, the load on the blade gradually increases. These factors influence the interference between vortex structures in the tip region, thereby altering the distribution of losses at the blade tip. In this section, the simulations are conducted for i=0°, 4°, 8°, respectively. Then, an analysis of the vortex structure with variations in the incidences is conducted. Subsequently, a quantitative assessment based on entropy generation reveals the impact mechanism of the incidence on the loss distribution in the blade tip flow field.

#### 3.3.1. The Vortex Structure

**Figure 10:**
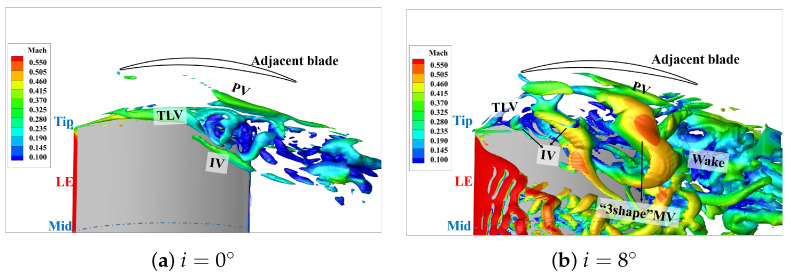
The transient vortex structure at $Ma_{1}=0.5,i=0{}^{\circ},8{}^{\circ}$ (iso-surface $Liutex_{mag}=8000$ contoured by Mach number).

Figure 10 presents the vortex structures at the incidence of 0° and 8°, respectively, and the comparison with the results at 4° from Figure 7b indicates a significant influence of the incidence on the vortex structures at the tip region.

Specifically, the TLV closely wraps around the vortex core before 70%C at i=0°. Subsequently, the leakage vortex begins to break down, and low-energy fluid converges near the suction surface, generating an IV around 65%C. However, the IV does not expand and interact with the PV or low-energy fluid. Therefore, there is no formation of the “3-shape” MV in the flow field. At the incidence of 8°, due to an increase in blade loading and an advanced position of the high loading, the TLV starts to deflect at 10%C and prematurely breaks at 15%C. The induced vortex generates at 15% and begins to extend at 50%C. Finally, it detaches prematurely from the suction surface driven by the surrounding low-energy fluid and PV, resulting in the early detachment of the “3-shape” MV. The spatial range of the “3-shape” MV is larger than that at at i=4°. Additionally, it quickly breaks after detaching from the trailing edge and mixes with the wake.

It can be observed that an increase in incidence leads to an increase in blade loading, causing the TLV deflecting into the passage and break earlier. This results in the premature generation of the IV, promoting the mixing of the IV with the passage vortex, and leading to the early formation of the “3-shape” MV.

#### 3.3.2. The Distribution and Quantification Analysis of the Tip Loss

**Figure 11:**
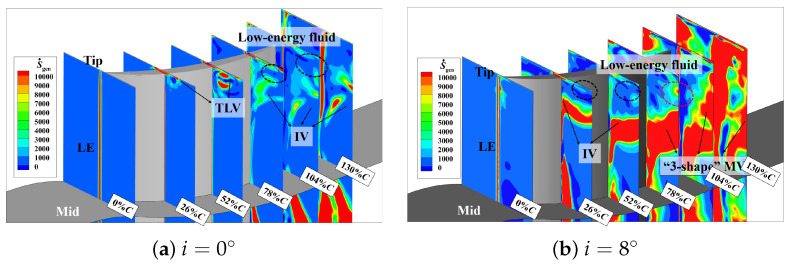
The distribution of the entropy generation rate at $Ma_{1}=0.5,i=0{}^{\circ},8{}^{\circ}$.

The entropy generation rate distribution in the tip region at i=0° and 8° is shown in Figure 11. At i=0°, the main source of secondary flow losses in the passage due to the TLV before 52%, while the mutual blending of low-energy fluid after this position. The generation location of IV is relatively posterior, and its interference with other vortex structures is limited, resulting in a smaller spanwise and tangential influence range. With an increase in incidence, it is evident that in the passage secondary flow, the range and intensity of losses caused by the IV gradually increase and move closer to the upstream region.

**Figure 12:**
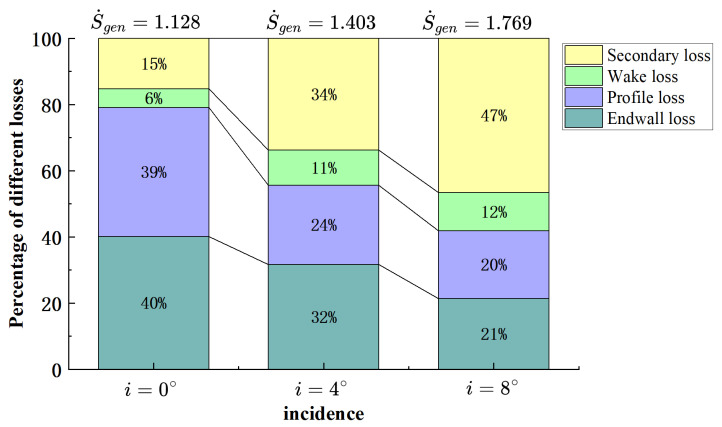
The proportion of four types of loss at different incidence.

Based on Equation (9), the entropy generation rates in the various loss source regions were integrated at i=0°,4°,8°, respectively. It should be noted that the integration regions vary with the operating conditions. In particular, as the incident angle increases, the boundary layer of the blade surface thickens, causing the integration region to grow from 1 mm to 3 mm from the blade surface. Meanwhile, the integration range of the wake region also widens according to the operating conditions. As is shown in Figure 12, it can be observed that the absolute values of losses in the tip region increase with the increase in the incidence. The main sources of losses in the tip region are from the endwall boundary layer and blade profile losses at i=0°, accounting for 40% and 39%, respectively, with wake and secondary flow losses accounting for only about 21%. With the increase in the incidence, the absolute value of endwall losses shows no significant variation, but both the absolute and relative values of blade profile losses gradually decrease. This is because at i=0°, the main stream on the blade surface is primarily attached to the suction surface. As the incidence increases, the airflow that was originally attached to the suction surface separates earlier, entering the blade passage, leading to a reduction in blade profile losses. The absolute and relative values of the wake losses increase with the increase in the incidence, but the magnitude is limited. This is because although the blade load continues to increase, most of the airflow at the suction surface trailing edge has already entered into space with the “3-shape” MV. As a result, the airflow mixing with the pressure surface trailing edge decreases.

The most significant change is observed in the losses induced by the tip passage secondary flow. At i=0, since the secondary flow in the passage mainly consists of the tip leakage vortex, and its breakup position is relatively far downstream, the resulting losses are relatively small. As the incidence increases to 4°, the influence of the IV inside the blade passage becomes dominant, leading to a sharp increase in the losses induced by the “3-shape” MV formed by the interaction with PV and low-energy fluid. With further increases in the incidence, the premature breakdown of the TLV results in an earlier generation of IV and the confluence point of the three vortices, leading to an expanded range and increased losses.

## 4. Conclusions

This paper focuses on a high-load compressor cantilevered stator cascade and obtains a high-fidelity result of the tip flow field used by the Enhanced Delay Detached Eddy Simulation (EDDES) method. Based on this, the correlation between flow-field losses and structural features is established according to the entropy production rate. The study explores the influence mechanism of variations in the incidence on tip losses. The main conclusions are as follows:

(1) By comparing the flow pattern visualizations and Mach number distribution out of the passage in the tip region between the experiment and numerical results, it can be concluded that the EDDES method exhibits higher accuracy in predicting the tip flow field compared to the RANS method. However, on the endwall, the limited streamlines of the EDDES results exhibit noticeable differences in the tangential direction compared to the oil flow patterns, indicating a deviation in the prediction of the tangential range of the leakage flow.
(2) There are four distinct vortex structures in the tip region: the tip leakage vortex, passage vortex, induced vortex, and “3-shape” mixed vortex. Among them, the induced vortex is crucial for the generation of the “3-shape” mixed vortex, and the induced vortex generates through the shear induced by the leakage vortex on the low-energy fluid near the suction surface.
(3) The sources of losses in the tip region include endwall loss, blade profile wake losses, and secondary flow loss. As the incidence increases, the absolute value of losses in the tip flow field increases. At i=0°, the percentage of endwall loss and blade profile loss is 79%, while the percentage of wake loss and secondary flow loss is 21%. As the incidence increases, the endwall loss remains relatively constant, the blade profile loss decreases due to the early separation on the suction surface, the wake loss shows a modest increase, and the secondary flow loss increases significantly. At i=8°, losses from the endwall, blade profile, wake, and secondary flow in the passage account for 21%, 20%, 12%, and 47%, respectively, with the secondary flow in the passage being the most significant contributor.

## Figures and Tables

**Table 1:** The main geometric parameters of the NPU01 cascade.

Parameters	Variable	Value	Unit
No. of blades	-	8	[-]
Blade chord length	*C*	65	mm
Aspect ratio	h/C	1.523	[-]
Blade solidity	τ	1.73	[-]
Tip clearance	δ	1	mm
Blade stagger angle	$\beta_{s}$	21.27	°
Blade inlet angle	$\beta_{1k}$	47.08	°
Blade outlet angle	$\beta_{2k}$	−1.98	°
Camber angle	θ	49.16	°
Design inlet Mach number	$Ma_{1d}$	0.4	[-]
Design flow incidence	$i_{d}$	0	°

**Table 2:** The inlet and outlet boundary condition.

Parameters	Parameter	Value
Inlet	Total pressure, Pa	110,865
	Total temperature, K	308.5
	Turbulence intensity, [-]	1.5∼2%
	Incidence, deg	0, 4, 8
Outlet	Pressure, Pa	97,482.5

**Table 3:** The grids in different regions.

Region	A	B	C
streamwise × spanwise × tangential	49×289×73	**H**: 217×289×49	25×289×73
		**O**: 353×289×41	

## Data Availability

The data that support the findings of this study are available from the corresponding author upon reasonable.
